# Supplementary material for: A Pilot Randomised Controlled Trial Evaluating a Regenerative Epithelial Suspension for Medium-Size Partial-Thickness Burns in Children: The BRACS Trial
Source: Eur Burn J. 2023 Mar 22;4(1):121–41. doi: 10.3390/ebj4010012 (PMC11571849; doi:10.3390/ebj4010012)
Supplement: Supplementary file 1 [file ebj-04-00012-s001.zip › ebj-2190353-supplementary.pdf]

Supplementary File: Bracs Trial Supplementary DATA.

Supplementary File S1. Scar thickness for participants < 8 years.

Scar Thickness	Silver Dressings		RES™/ Biobrane®		Biobrane only®	
	Median (IQR)	Mean (SD)	Median (IQR)	Mean (SD)	Median (IQR)	Mean (SD)
**3-month Follow-up (n)**	5		4		5	
Scar thickness (mm)	1.37 (0.96-1.97)	1.45 (0.64)	1.16 (1.00-1.31)	1.16 (0.19)	1.14 (0.98-5.02)	2.62 (2.70)
**6-month Follow-up (n)**	7		4		5	
Scar thickness (mm)	1.97 (1.28-2.43)	1.88 (0.61)	1.19 (1.14-1.27)	1.20 (0.07)	1.75 (1.31-5.72)	3.16 (3.30)
**12-month Follow-up (n)**	5		4		4	
Scar thickness (mm)	1.32 (0.85-2.92)	1.77 (1.22)	1.08 (0.97-1.49)	1.18 (0.29)	1.59 (1.35-2.94)	1.96 (0.94)

Abbreviation: IQR: interquartile range; **SD:** standard deviation; **RES™**: Regenerative Epidermal Suspension.

Supplementary File S2. Scar colorimetry for participants < 8 years.

Scar Colorimetry	Silver Dressings		RES™ / Biobrane®		Biobrane only®	
	**Median (IQR)**	**Mean (SD)**	**Median (IQR)**	**Mean (SD)**	**Median (IQR)**	**Mean (SD)**
**3-month Follow-up(*n*)**	7		5		5	
L^*_{Scar}	36.51 (16.85-40.24)	30.43 (12.45)	37.42 (20.23-54.38)	37.33 (19.77)	29.70 (27.20-34.62)	30.67 (4.22)
L^*_{Normal}	44.151 (33.76-48.81)	41.54 (10.35)	34.77 (18.82-47.98)	33.67 (17.27)	37.25 (33.87-39.18)	36.67 (3.08)
a^*_{Scar}	19.84 (11.86-33.62)	22.92 (12.58)	21.02 (12.89-26.12)	19.81 (7.93)	20.49 (16.28-25.89)	20.97 (5.77)
a^*_{Normal}	17.78 (9.47-19.07)	16.17 (6.13)	17.99 (14.55-25.07)	17.99 (5.54)	13.93 (13.18–21.69)	16.73 (4.54)
b^*_{Scar}	12.11 (8.38-15.45)	11.86 (8.38)	7.57 (4.39-12.33)	8.20 (5.04)	11.61 (6.93-16.33)	11.62 (4.79)
b^*_{Normal}	19.29 (13.74-21.04)	18.07 (3.64)	9.84 (7.89-18.78)	12.63 (5.85)	14.88 (7.78-18.72)	13.57 (5.86)
**6-month Follow-up(*n*)**	6		5		5	
L^*_{Scar}	40.36 (28.80-45.57)	38.47 (10.53)	38.32 (30.02-40.31)	35.79 (5.76)	25.81 (18.20-35.69)	26.72 (9.78)
L^*_{Normal}	41.23 (31.16-54.93)	41.93 (12.78)	37.05 (29.97-40.19)	35.47 (5.87)	35.08 (31.59-40.24)	35.75 (4.85)
a^*_{Scar}	21.29 (11.36-25.90)	19.75 (8.30)	22.08 (15.69-27.09)	21.53 (6.16)	20.67 (14.13-23.93)	19.36 (6.00)
a^*_{Normal}	14.63 (11.7-22.48)	16.36(2.45)	14.42 (12.71-18.31)	15.29 (3.21)	15.34 (11.57–18.46)	15.08 (3.59)
b^*_{Scar}	15.49 (12.01-20.50)	16.41 (5.02)	11.15 (8.65-16.80)	12.41 (5.77)	12.51 (9.11-16.52)	12.75 (4.06)
b^*_{Normal}	15.61 (14.55-17.70)	16.33 (2.45)	15.44 (11.48-17.74)	14.78 (4.03)	19.09 (9.32-25.95)	17.63 (9.37)
**12-month Follow-up(*n*)**	4		4		5	
L^*_{Scar}	32.10 (15.61-40.90)	29.54 (14.06)	35.78 (15.80-41.26)	30.95 (14.46)	39.13 (30.05-45.00)	38.06 (8.18)
L^*_{Normal}	29.84 (26.60-40.89)	32.44 (8.12)	32.18 (16.53-46.78)	31.53 (16.10)	43.73 (33.80-46.58)	41.37 (7.26)
a^*_{Scar}	14.23 (9.91-18.70)	14.28 (4.62)	13.35 (10.05-16.84)	13.41 (3.55)	15.12 (13.07-17.57)	15.25 (2.34)
a^*_{Normal}	13.53 (12.57-14.38)	13.49 (0.98)	13.24 (11.17-15.76)	13.24 (2.57)	11.23 (10.16–15.88)	12.43 (3.40)
b^*_{Scar}	13.94 (10.88-16.85)	13.89 (3.09)	14.29 (8.88-16.87)	13.35 (4.30)	9.35 (3.28-18.11)	10.24 (7.84)
b^*_{Normal}	16.87 (15.02-18.27)	16.72 (1.73)	15.61 (10.91-18.26)	14.93 (3.95)	14.85 (12.77-18.94)	15.52 (3.33)

Abbreviation: IQR: Interquartile range; **SD**: Standard deviation; **RES™**: Regenerative Epidermal Suspension; **L***: Lightness; **a***: erythema; **b***: pigmentation.

Supplementary File S3. Clinician report of scar severity with OSAS for participants < 8 years.

Clinician OSAS < 8 years	Silver Dressings		RES™ /Biobrane®		Biobrane only®	
	Median (IQR)	Mean (SD)	Median (IQR)	Mean (SD)	Median (IQR)	Mean (SD)
**3-month Follow-up(*n*)**	7		6		5	
Scar Age post injury (months)	2.00(2.00-400)	2.71(0.95)	3.00(2.00-6.00)	4.67(5.09)	2.00(2.00-3.00)	2.40(0.55)
Vascularity	2.00(1.00-4.00)	2.57(1.72)	1.50(1.00-2.25)	1.67(0.82)	4.00(2.00-8.50)	5.00(3.39)
Pigmentation	3.00(2.00-7.00)	4.57(2.99)	4.00(1.00-4.00)	3.00(1.50)	4.00(2.50-5.00)	3.80(1.48)
Thickness	3.00(1.00-8.00)	3.57(3.16)	1.00(1.00-1.00)	1.00(0.00)	4.00(1.50-7.50)	4.40(3.51)
Relief	4.00(1.00-7.00)	3.57(2.70)	1.00(1.00-1.25)	1.17(0.41)	3.00(2.00-8.00)	4.60(3.13)
Pliability	2.00(1.00-7.00)	3.43(3.60)	1.00(1.00-1.00)	1.00(0.00)	3.00(1.50-6.00)	3.60(2.70)
Overall Opinion	3.00(2.00-4.00)	3.57(2.23)	4.00(2.75-4.00)	3.50(0.84)	4.00(2.50-7.00)	4.60(2.30)
**6-month Follow-up(*n*)**	7		4		5	
Scar Age post injury (months)	5.00(5.00-6.00)	5.43(0.54)	5.00(5.00-5.75)	5.25(0.50)	6.00(5.00-7.00)	6.00(1.00)
Vascularity	3.00(1.00-3.00)	2.43(1.13)	1.50(1.00-2.00)	1.50(0.58)	4.00(2.50-7.00)	4.60(2.30)
Pigmentation	3.00(2.00-5.00)	3.57(2.23)	2.00(1.00-6.75)	3.25(3.30)	2.00(2.00-2.00)	2.00(0.00)
Thickness	4.00(1.00-6.00)	3.71(3.04)	1.00(1.00-1.00)	1.00(0.00)	3.00(2.00-8.00)	4.60(3.51)
Relief	3.00(1.00-6.00)	3.29(2.36)	1.00(1.00-1.00)	1.00(0.00)	4.00(1.50-8.50)	4.80(3.56)
Pliability	2.00(1.00-5.00)	3.14(2.67)	1.00(1.00-1.75)	1.25(0.50)	3.00(1.50-6.50)	3.80(2.78)
Overall Opinion	3.00(2.00-6.00)	3.57(2.23)	2.00(1.25-5.00)	2.75(2.22)	4.00(2.50-7.50)	4.80(2.59)
**12-month Follow-up(*n*)**	5		4		4	
Scar Age post injury (months)	12.00(11.50-12.00)	11.80(0.45)	11.50(11.00-12.00)	11.50(0.58)	12.00(11.25-12.00)	11.75(0.50)
Vascularity	1.00(1.00-2.00)	1.40(0.55)	1.00(1.00-1.75)	1.00(0.50)	1.50(1.00-5.00)	2.50(2.38)
Pigmentation	2.00(1.50-6.50)	3.60(3.65)	3.00(1.25-6.25)	3.50(2.65)	2.50(2.00-4.50)	3.00(1.41)
Thickness	2.00(1.00-5.00)	2.80(2.95)	1.00(1.00-1.75)	1.25(0.50)	3.00(1.25-7.00)	3.75(3.10)
Relief	1.00(1.00-4.00)	2.40(2.61)	0.50(0.00-1.00)	0.50(0.58)	2.50(1.25-8.25)	4.00(4.08)
Pliability	1.00(1.00-4.00)	2.20(2.68)	1.00(1.00-1.00)	1.00(0.00)	1.50(1.00-7.25)	3.25(3.86)
Overall Opinion	2.00(2.00-5.50)	3.40(3.13)	2.00(1.25-2.75)	2.00(0.82)	2.50(2.00-6.75)	3.75(2.87)

Abbreviation: IQR: Interquartile range; **SD**: Standard deviation; **RES™**: Regenerative Epidermal Suspension; **OSAS**: Observer Scar Assessment Scale.

Supplementary File S4. Parent/guardian report of scar severity with POSAS for participants < 8 years.

Parent/Guardian POSAS < 8years	Silver Dressings		RES™ /Biobrane®		Biobrane only®	
	Median (IQR)	Mean (SD)	Median (IQR)	Mean (SD)	Median (IQR)	Mean (SD)
**3-month Follow-up (*n*)**	6		6		5	
Scar Age post injury (months)	2.50 (2.00-4.00)	2.83 (0.98)	2.50 (2.00-3.00)	2.50 (0.55)	2.00 (2.00-3.00)	2.40 (0.55)
Pain	1.00 (1.00-1.00)	1.00 (0.00)	1.00 (1.00-1.25)	1.17 (0.41)	1.00 (1.00-2.50)	1.60 (0.90)
Itch	1.00 (1.00-5.75)	2.83 (2.99)	1.50 (1.00-2.75)	2.00 (1.55)	3.00 (1.50-9.00)	4.80 (4.03)
Colour	9.00 (4.50-10.00)	7.67 (3.01)	6.00 (5.50-6.50)	6.00 (1.27)	9.00 (6.00-9.50)	8.00 (1.87)
Stiffness	4.00 (1.00-8.00)	4.33 (3.67)	3.00 (1.00-6.50)	3.67 (2.81)	5.00 (3.00-9.50)	6.00 (3.32)
Thickness	5.50 (1.00-9.25)	5.33 (4.13)	2.00 (1.00-6.50)	3.33 (3.01)	8.00 (2.50-10.00)	6.60 (3.98)
Irregular	5.00 (1.00-10.00)	5.33 (4.46)	5.50 (1.75-8.00)	5.00 (2.97)	8.00 (4.00-10.00)	7.20 (3.03)
Overall Opinion	7.00 (2.75-10.00)	6.50 (3.45)	5.50 (4.50-7.50)	5.83 (2.04)	9.00 (4.00-10.00)	7.40 (3.13)
POSAS Score	26.50 (13.50-40.00)	26.50 (14.00)	19.00 (13.50-29.75)	21.17 (10.15)	40.00 (19.50-46.00)	34.20 (14.13)
**6-month Follow-up (*n*)**	7		6		5	
Scar Age post injury (months)	5.00 (5.00-6.00)	5.43 (0.54)	5.50 (5.00-6.25)	5.67 (0.82)	6.00 (5.00-6.50)	5.80 (0.84)
Pain	1.00 (1.00-1.00)	1.00 (0.00)	1.00 (1.00-1.00)	1.00 (0.00)	1.00 (1.00-2.00)	1.40 (0.55)
Itch	3.00 (1.00-6.00)	3.43 (2.64)	1.00 (1.00-1.00)	1.00 (0.00)	3.00 (1.00-7.00)	3.80 (3.11)
Colour	3.00 (2.00-9.00)	5.14 (3.67)	3.00 (2.75-5.00)	3.50 (1.23)	6.00 (4.00-6.50)	5.40 (1.52)
Stiffness	2.00 (1.00-6.00)	4.00 (3.42)	1.00 (1.00-2.25)	1.50 (0.84)	4.00 (2.00-6.50)	4.20 (2.39)
Thickness	4.00 (1.00-8.00)	4.57 (3.55)	1.00 (1.00-3.00)	1.67 (1.03)	6.00 (2.50-7.50)	5.20 (2.78)
Irregular	2.00 (1.00-10.00)	4.57 (4.08)	1.00 (1.00-1.50)	1.33 (0.82)	6.00 (2.50-7.50)	5.20 (2.59)
Overall Opinion	5.00 (3.00-10.00)	5.86 (3.44)	2.00 (1.00-3.00)	2.00 (0.89)	6.00 (3.00-7.00)	5.20 (2.05)
POSAS Score	17.00 (11.00-37.00)	22.71 (13.91)	9.00 (2.00-1.00)	7.67 (4.80)	26.00 (14.50-35.50)	25.20 (11.21)
**12-month Follow-up (*n*)**	7		5		4	
Scar Age post injury (months)	12.00 (11.00-12.00)	11.71 (0.49)	12.00 (11.00-12.50)	11.80 (0.84)	12.00 (11.25-12.00)	11.75 (0.50)
Pain	1.00 (1.00-1.00)	1.00 (0.00)	1.00 (1.00-1.00)	1.00 (0.00)	1.00 (1.00-1.00)	1.00 (0.00)
Itch	1.00 (1.00-4.00)	2.14 (1.46)	1.00 (1.00-1.00)	1.00 (0.00)	2.00 (1.25-3.50)	2.25 (1.26)
Colour	3.00 (1.00-7.00)	4.00 (3.37)	2.00 (1.00-4.00)	2.40 (1.67)	5.50 (4.00-8.50)	6.00 (2.45)
Stiffness	3.00 (1.00-5.00)	3.71 (2.87)	1.00 (1.00-2.00)	1.40 (0.55)	2.50 (2.00-5.25)	3.25 (1.89)
Thickness	3.00 (1.00-7.00)	4.14 (3.19)	1.00 (1.00-3.00)	1.80 (1.30)	4.50 (3.00-7.50)	5.00 (2.45)
Irregular	3.00 (1.00-6.00)	3.57 (2.94)	1.00 (1.00-3.00)	1.80 (1.30)	4.50 (3.00-7.50)	5.00 (2.45)
Overall Opinion	3.00 (1.00-6.00)	3.86 (2.97)	2.00 (1.00-3.50)	2.20 (1.64)	3.50 (3.00-5.50)	4.00 (1.41)
POSAS Score	14.00 (6.00-27.00)	18.00 (14.06)	9.00 (4.00-13.00)	8.60 (5.18)	22.50 (14.75-30.25)	22.50 (8.27)

Abbreviation: IQR: Interquartile range; **SD**: Standard deviation; **RES™**: Regenerative Epidermal Suspension; **POSAS**: Patient Observer Scar Assessment Scale.

Supplementary File S5. Parent/guardian report of scar specific health related quality of life with BBSIP for participants < 8 years old.

Parent/Guardian BBSIP <8 years	Silver Dressings		RES™ /Biobrane®		Biobrane only®	
	Median (IQR)	Mean (SD)	Median (IQR)	Mean (SD)	Median (IQR)	Mean (SD)
**3-month Follow-up (*n*)**	7		6		4	
Scar age post injury(months)	3.00 (3.00-4.00)	3.29 (0.49)	3.00 (2.75 -3.25)	3.00 (0.63)	3.00 (300-3.00)	3.00 (0.00)
Overall impact of burns	*2.13 (1.63-3.13)*	*2.21 (0.83)*	*1.44 (1.34-2.00)*	*1.63 (0.43)*	*2.63 (1.50-2.82)*	*2.31 (0.80)*
Sensory Frequency	1.67 (1.00-3.33)	2.14 (1.15)	1.50 (1.00-2.08)	1.67 (0.87)	2.67 (1.25-3.33)	2.42 (1.13)
Sensory Sensitivity	2.00 (1.00-4.00)	2.29 (2.06)	1.00 (0.75-2.00)	1.50 (1.76)	4.50 (1.50-6.00)	4.00 (2.45)
Mobility	1.25 (1.25-2.00)	1.54 (0.53)	1.25 (1.19-2.63)	1.79 (1.05)	2.25 (1.44-2.69)	2.13 (0.66)
Daily Living	1.56 (1.11-2.33)	1.76 (0.58)	1.33 (1.08-2.50)	1.76 (1.02)	2.00 (1.17-2.75)	1.97 (0.82)
Impact of scar daily routine	2.00 (1.00-300)	2.29 (1.80)	1.00 (1.00-1.00)	1.00 (0.00)	2.00 (1.25-2.00)	1.75 (0.50)
Impact of scar on developing independence or new skills	1.00 (1.00-1.00)	1.00 (0.00)	1.00 (1.00-1.00)	1.00 (0.00)	1.00 (1.00-1.75)	1.25 (0.50)
Friendship and social interaction	1.00 (1.00-2.00)	1.62 (1.25)	1.00 (1.00-1.08)	1.05 (0.14)	1.50 (1.0-2.00)	1.42 (0.32)
Appearance	2.33 (1.00-4.00)	2.33 (1.37)	2.00 (1.25-2.92)	2.11 (0.98)	2.33 (1.25-2.67)	2.08 (0.79)
Child bothered by scar appearance	1.00 (1.00-1.00)	1.00 (0.58)	1.00 (1.00-1.50)	1.33 (0.82)	1.50 (1.00-2.00)	1.50 (0.58)
Emotional reactions	1.14 (1.14-1.43)	1.29 (0.27)	1.14 (1.14-1.32)	1.27 (0.29)	1.86(1.29-2.11)	1.75 (0.44)
Physical symptoms	2.29 (1.29-3.14)	2.20 (0.86)	1.43 (1.39-2.04)	1.62 (0.36)	2.57 (2.18-3.29)	2.68 (0.59)
Parent worry	3.33 (1.00-4.33)	2.95 (1.70)	1.67 (1.25-2.17)	1.72 (0.57)	1.67 (1.08-3.25)	2.00 (1.19)
Parent impact	1.20 (1.00-3.20)	1.94 (1.08)	1.00 (1.00-1.00)	1.00 (0.00)	1.70 (1.15-2.25)	1.70 (0.58)
**6-month Follow-up (*n*)**	7		6		5	
Scar age post injury(months)	6.00 (6.00-6.00)	6.14 (0.38)	6.00 (6.00-6.00)	6.00 (0.00)	7.00 (5.50-7.00)	6.40 (0.89)
Overall impact of burns	*1.63 (1.38-1.75)*	*1.55 (0.31)*	*1.00 (1.00-1.03)*	*1.02 (0.05)*	*1.38 (1.13-2.38)*	*1.68 (0.70)*
Sensory Frequency	1.67 (1.00-2.33)	1.76 (0.63)	1.00 (1.00-1.08)	1.06 (0.14)	1.67 (1.00-2.83)	1.87 (0.93)
Sensory Sensitivity	0.00 (0.00-1.00)	0.43 (0.53)	1.00 (0.75-1.50)	1.17 (0.98)	1.00 (0.00-2.00)	1.00 (1.00)
Mobility	1.00 (1.00-1.25)	1.11 (0.34)	1.25 (1.19-1.38)	1.29 (0.25)	1.25 (1.00-1.50)	1.25 (0.25)
Daily Living	1.56 (1.00-1.56)	1.35 (0.27)	1.00 (1.00-1.00)	1.00 (0.00)	1.78 (1.56-2.17)	1.84 (0.31)
Impact of scar daily routine	1.00 (1.00-1.00)	1.00 (0.00)	1.00 (1.00-1.00)	1.00 (0.00)	1.00 (1.00-1.50)	1.20 (0.45)
Impact of scar on developing independence or new skills	1.00 (1.00-1.00)	1.00 (0.00)	1.00 (1.00-1.00)	1.00 (0.00)	1.00 (1.00-1.00)	1.00 (0.00)
Friendship and social interaction	1.00 (1.00-1.33)	1.14 (0.18)	1.00 (1.00-1.00)	1.00 (0.00)	1.00 (1.00-1.67)	1.27 (0.43)
Appearance	1.00 (1.00-2.00)	1.29 (0.78)	1.00 (1.00-1.17)	1.11 (0.27)	1.33 (1.00-1.67)	1.33 (0.33)
Child bothered by scar appearance	1.00 (1.00-1.00)	0.86 (0.38)	1.00 (1.00-1.00)	1.00 (0.00)	1.00 (1.00-2.00)	1.40 (0.55)
Emotional reactions	1.14 (1.14-1.29)	1.04 (0.47)	1.14 (1.11-1.18)	1.14 (0.09)	1.43 (1.14-2.36)	1.69 (0.64)
Physical symptoms	1.86 (1.43-2.43)	1.92 (0.67)	1.29 (1.11-1.36)	1.26 (0.19)	2.00 (1.43-2.57)	2.00 (0.71)
Parent worry	1.33 (1.00-1.67)	1.48 (0.60)	1.00 (1.00-1.08)	1.06 (0.14)	1.33 (1.00-2.33)	1.60 (0.83)
Parent impact	1.00 (1.00-1.20)	1.11 (0.16)	1.00 (1.00-1.00)	1.00 (0.00)	1.00 (1.00-1.50)	1.20 (0.28)
**12-month Follow-up (*n*)**	7		5		4	

Scar age post injury(months)	12.00 (12.0-13.00)	12.29 (0.49)	12.00 (12.00-13.00)	12.40 (0.55)	12.00 (12.00-12.75)	12.25 (0.50)
Overall impact of burns	*1.13 (1.00-3.00)*	*1.71 (0.95)*	*1.00 (1.00-1.00)*	*1.00 (0.00)*	*1.38 (1.09-1.56)*	*1.34 (0.26)*
Sensory Frequency	1.00 (1.00-2.00)	1.38 (0.68)	1.00 (1.00-1.00)	1.00 (0.00)	1.50 (1.00-2.25)	1.58 (0.69)
Sensory Sensitivity	1.00 (0.00-3.00)	1.29 (1.60)	0.00 (0.0-1.00)	0.40 (0.55)	0.50 (0.00-2.50)	1.00 (1.41)
Mobility	1.25 (1.00-2.50)	2.00 (1.44)	1.00 (1.0-1.25)	1.10 (0.14)	1.13 (1.00-1.63)	1.25 (0.35)
Daily Living	1.00 (1.00-2.11)	1.84 (1.51)	1.00 (1.00-1.00)	1.00 (0.00)	1.11 (1.00-1.47)	1.19 (0.26)
Impact of scar daily routine	1.00 (1.00-2.00)	1.86 (1.86)	1.00 (1.00-1.00)	1.00 (0.00)	1.00 (1.00-1.00)	1.00 (0.00)
Impact of scar on developing independence or new skills	1.00 (1.00-2.00)	1.71 (1.50)	1.00 (1.00-1.00)	1.00 (0.00)	1.00 (1.00-1.00)	1.00 (0.00)
Friendship and social interaction	1.00 (1.00-2.00)	1.70 (1.50)	1.00 (1.00-1.00)	1.00 (0.00)	1.00 (1.00-1.25)	1.08 (0.17)
Appearance	1.00 (1.00-2.00)	1.57 (0.89)	1.00 (1.00-1.17)	1.07 (0.15)	1.33 (1.08-1.58)	1.33 (0.27)
Child bothered by scar appearance	1.00 (1.00-1.00)	1.14 (0.38)	1.00 (1.00-1.00)	1.00 (0.00)	1.00 (1.00-1.75)	1.25 (0.50)
Emotional reactions	1.14 (1.14-1.29)	1.25 (0.21)	1.14 (1.14-1.14)	1.14 (0.00)	1.21 (1.14-1.39)	1.25 (0.14)
Physical symptoms	1.71 (1.00-2.00)	1.86 (0.98)	1.14 (1.14-1.43)	1.23 (0.19)	2.00 (1.75-2.14)	1.96 (0.21)
Parent worry	1.33 (1.00-2.33)	1.57 (0.79)	1.00 (1.00-1.17)	1.07 (0.15)	1.17 (1.00-1.58)	1.25 (0.32)
Parent impact	1.00 (1.00-1.60)	1.20 (0.35)	1.00 (1.00-1.00)	1.00 (0.00)	1.00 (1.00-1.00)	1.00 (0.00)

Abbreviation: IQR: Interquartile range; **SD**: Standard deviation; **RES™**: Regenerative Epidermal Suspension.

Supplementary File S6. Paediatric Health Related Quality Of Life Based On Parent Reported Chu9d.

Parent/Guardian CHU9D	Silver dressings	RES™ /Biobrane®	Biobrane® only
**3-month Follow up (*n*)**	7	6	5
Median	0.94	1.00	0.75
IQR	0.76-1.00	0.77-1.00	0.44-0.93
Mean	0.88	0.87	0.70
Total Range	0.35	0.68	0.64
**6-month Follow up (*n*)**	7	6	5
Median	0.94	0.94	0.97
IQR	0.90-1.00	0.78-1.00	0.55-1.00
Mean	0.94	0.91	0.81
Total Range	0.16	0.22	0.53
**12-month Follow up(*n*)**	7	5	4
Median	1.00	1.00	0.98
IQR	0.92-1.00	0.89-1.00	0.85-1.00
Mean	0.98	0.96	0.95
Total Range	0.09	0.22	0.19

Abbreviation: IQR: Interquartile range; **CHU9D:** Child Health Utility.

Supplementary File S7. Health resource utilisation.

**Health Resource Utilisation** (*n*)	**Silver dressings** 8	**RES™ /Biobrane®** 7	**Biobrane®** 7
**Intervention Cost**			
Initial Dressing Application			
Median	$291.30	$9262.00	$667.92
IQR	$194.02-$438.05	$8830.10-$21280.70	$498.97-$1361.72
Mean	$344.23	$13464.94	$847.15
Total Range	$552.90	$13525.19	$1608.14
Dressing Change			
Median	$645.87	$63.91	$385.38
IQR	$278.70-$933.43	$28.34-$891.83	$261.33-$666.81
Mean	$612.65	$358.52	$521.05
Total Range	$1202.25	$906.51	$1268.67
**Hospitalisation Setting**			
Emergency Department			
Median	$851.04	$642.29	$825.42
IQR	$548.62-$1414.20	$367.99 - $1270.38	$443.87 - $1495.54
Mean	$936.53	$770.60	$1084.42
Total Range	$1362.27	$1152.79	$2860.90
Inpatient			
Median	$32581.93	$21707.34	$47058.00
IQR	$6737.32 - $73070.61	$4424.21-$76438.30	$1466.41-$102527.18
Mean	$3877.28	$42363.14	$56236.73
Total Range	$95006.06	$128111.42	$97086.05
Outpatient			
Median	$314.16	$314.57	$314.16
IQR	$304.87 - $444.67	$304.87-$424.34	$304.87 - $380.71
Mean	$370.23	$353.39	$336.67
Total Range	$852.22	$850.02	$388.53

Abbreviation: IQR: Interquartile range.
